# Effect of mental stress on dynamic electrophysiological properties of the endocardium and epicardium in humans

**DOI:** 10.1016/j.hrthm.2015.08.011

**Published:** 2016-01

**Authors:** Malcolm C. Finlay, Pier D. Lambiase, Ron Ben-Simon, Peter Taggart

**Affiliations:** * University College London, Queen Mary University of London & St Bartholomew’s Hospital, London, United Kingdom; † Barts Heart Centre, St Bartholomew’s Hospital, London, United Kingdom,; ‡ University College London, University College London Hospitals NHS Foundation Trust, London, United Kingdom

**Keywords:** **APD**, action potential duration, **ARI**, activation recovery interval, **ERP**, effective refractory period, **LV**, left ventricular, **RV**, right ventricle/ventricular, Arrhythmia, Mental stress, Restitution, Dispersion of repolarization, Human electrophysiology

## Abstract

**Background:**

Striking temporal associations exist between ventricular arrhythmia and acute mental stress, for example, during natural disasters, or defibrillator shocks associated with stressful events. We hypothesized that electrophysiological changes in response to mental stress may be exaggerated at short coupling intervals and hence relevant to arrhythmia initiation.

**Objective:**

The aim of this study was to determine the dynamic response in human electrophysiology during mental stress.

**Methods:**

Patients with normal hearts and supraventricular tachycardia underwent electrophysiological studies avoiding sedation. Conditions of relaxation and stress were induced with standardized psychometric protocols (mental arithmetic and anger recall) during decremental S_1_S_2_ right ventricular (RV) pacing. Unipolar electrograms were acquired simultaneously from the RV endocardium, left ventricular (LV) endocardium (LV endo), and epicardium (LV epi), and activation-recovery intervals (ARIs) computed.

**Results:**

Twelve patients ( 9 women; median age 34 years) were studied. During stress, effective refractory period (ERP) reduced from 228 ± 23 to 221 ± 21 ms (*P* < .001). ARIs reduced during mental stress (*P* < .001), with greater reductions in LV endocardium than in the epicardium or RV endocardium (LV endo −8 ms; LV epi −5 ms; RV endo −4 ms; *P* < .001). Mental stress depressed the entire electrical restitution curve, with minimal effect on slope. A substantial reduction in minimal ARIs on the restitution curve in LV endo occurred, commensurate with the reduction in ER*P* (LV endo ARI 195 ± 31 ms at rest to 182 ± 32 ms during mental stress; *P* < .001). Dispersion of repolarization increased sharply at coupling intervals approaching ERP during stress but not at rest.

**Conclusion:**

Mental stress induces significant electrophysiological changes. The increase in dispersion of repolarization at short coupling intervals may be relevant to observed phenomena of arousal-associated arrhythmia.

## Graphical abstract

**Figure:**
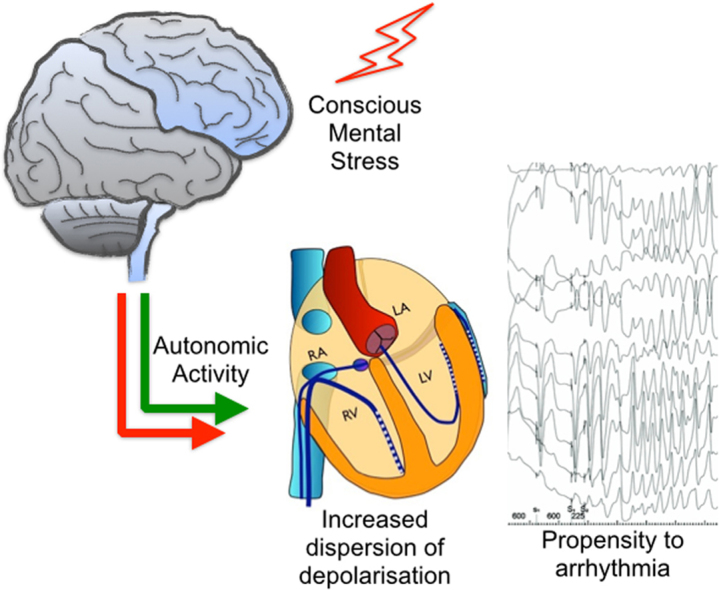

## Introduction

The ability of conscious mental activity to influence the risk of cardiac arrhythmia has been well recognized. Controlled data have showed implantable cardioverter-defibrillator shocks to be temporally associated with periods of mental stress.^1^, ^2^ In a series of elegant studies, mental stress increased T-wave alternans and decreased arrhythmia threshold in a series of ischemic heart disease patients with implantable cardioverter-defibrillators.^1^, ^3^ Furthermore, previous investigators have documented increased arrhythmogenicity in animal models during mental stress.^4^ These studies have confirmed a clear link between conscious activity and the risk of arrhythmia, but there are few data documenting the direct effects of such activity on cardiac ventricular electrophysiology.

Taggart et al^5^ demonstrated the shortening of the monophasic action potential duration (APD) in human ventricles in response to isoprenaline. A steepening of APD electrical restitution was also observed, which may imply an increased susceptibility to arrhythmia. Recently, our group demonstrated a decrease in the activation recovery interval (ARI; a well-validated surrogate of APD derived from unipolar electrograms) in response to fear independent of heart rate changes.^6^ However, dynamic changes in electrophysiology due to mental stress may be even more relevant to the initiation of arrhythmia-clinical arrhythmia often commences with a premature extrasystole. It has been hypothesized that mental stress can contribute to arrhythmia by increasing heterogeneity of repolarization and activation (or conduction velocity) throughout the heart. These effects may amplify preexisting heterogeneity especially in the context of ischemic and structural heart disease and thus lead to conduction wavebreak and arrhythmogenesis.

We hypothesized that electrophysiological changes in response to mental stress may become markedly exaggerated at short coupling intervals and hence relevant to arrhythmia initiation by premature beats. This was tested by performing electrophysiological studies on patients with normal ventricles at rest and during a controlled mental stress protocol.

## Methods

### Catheter placement and clinical studies

The protocol was performed in patients with structurally normal hearts undergoing cardiac electrophysiological studies for the diagnosis and ablation of supraventricular tachycardia. All patients had normal resting electrocardiograms, echocardiograms, and cardiac examinations. The study was approved by the ethics committee of University College London Hospitals (UCL REC no. 10/H7015/19) and conformed to the standards set by the Declaration of Helsinki. All subjects gave prior written informed consent; specifically they were informed that they would be asked to undergo mental arithmetic with firm encouragement and to recall stressful past events as part of the study. Studies were performed with a local anesthetic only in the postabsorptive state. Antiarrhythmic drugs were discontinued for 5 days preprocedure.

**Figure 1:**
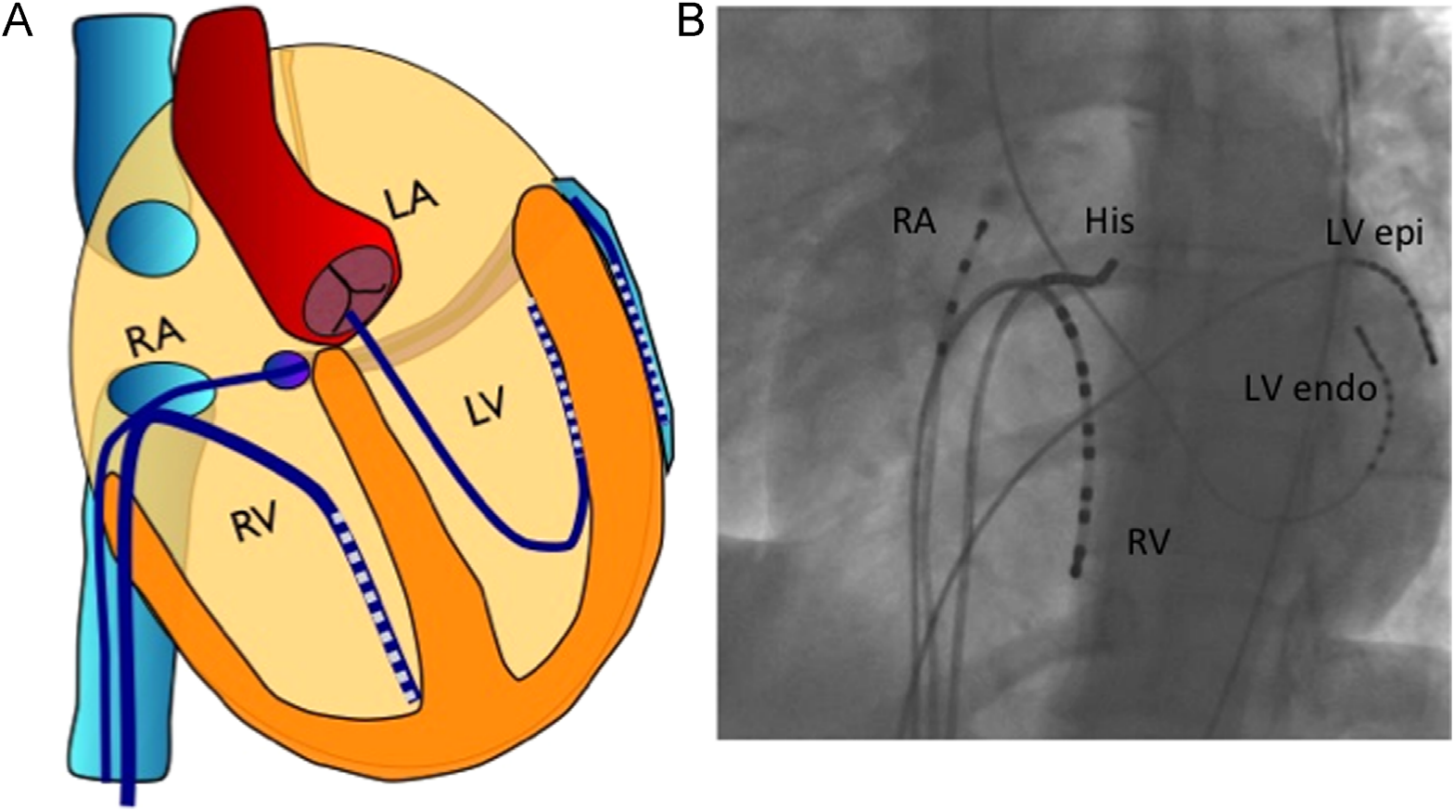
Catheter positions for in vivo studies. Schematic (**A**) and x-ray fluoroscopy (**B**) catheter positions are shown. endo = endocardial; epi = epicardial; LA = left atrium; LV = left ventricle; RA = right atrium; RV = right ventricle.

Catheters were placed via venous sheaths (6F to 8F), with 4F arterial access (left ventricular [LV] retrograde catheter). All patients received 5000 units of unfractionated heparin. Decapolar catheters were placed in an epicardial coronary vein (great cardiac/middle or lateral vein) via the coronary sinus, retrogradely within the LV cavity adjacent to the epicardial catheter (2-mm electrode spacing, Pathfinder, St Jude Medical Inc, St Paul, MN) and at the right ventricular (RV) apex (2-5-2 mm electrode spacing, Pathfinder; Figure 1). X-ray fluoroscopy was used to guide catheter electrode placement. A reference anodal electrode was placed in the inferior vena cava. Further catheters were placed according to clinical requirements following the research protocol. Electrograms were digitized and recorded at 1000 Hz (Bard Clearsign, CR Bard, NJ, USA, MN). Surface 12-lead electrocardiograms were recorded throughout the study. Oxygen saturations were measured continuously using pulse oximetry, and blood pressure was measured noninvasively at 5-minute intervals. The research study was performed before patients undergoing a clinical electrophysiological study.

Following the clinical procedure, venous and arterial sheaths were removed and hemostasis achieved with manual pressure.

### Restitution pacing protocol

A S_1_S_2_ restitution study was conducted using programmed electrical stimulation. Following 3 minutes of steady-state pacing at the RV apex at a cycle length of 600 ms, a drivetrain of 10 beats was followed by an extrastimulus (S_2_). The S_1_S_2_ interval was decremented by 20–300 ms and thereafter in 5-ms intervals until effective refractory period (ERP) was reached. The ERP was found by increasing coupling intervals (CIs) by 8 ms and then decrementing further by 2-ms intervals.

### Mental stress studies

Programmed electrical stimulation was performed during an active relaxation protocol and again during mental stress in the manner described by Burg et al.^7^ During active relaxation, subjects were asked to think of a situation that they found subjectively relaxing and were asked to imagine themselves being in that situation. Laboratory lighting was turned down to a minimum and staff remained silent during this stage of the study. Gentle prompts in a clam and reassuring manner were used to encourage the subject’s active memory of the situation.

Mental stress was induced by 2 methods: mental arithmetic (with harsh encouragement) and anger recall. Data are presented from the activity that provoked the greatest stress response. Laboratory lighting was returned to full during the stress protocol. Mental arithmetic involved subtraction of serial 7 seconds from a 4-digit number. Strong encouragement was given to maintain subjects’ interest and concentration. If the subject was willing, anger recall was subsequently performed. Subjects were asked to remember a recent situation that made them angry. They were asked to relive that situation and tell the investigator as if describing that situation later that same day to a close friend. The investigator then attempted to mildly provoke further reaction with awkward questioning. Subjective stress was recorded on a Likert scale for each protocol, which has been shown to accurately correlate with physiological measures of stress.^8^ Intrinsic heart rate and blood pressure were taken as objective measures of stress response. Periods of sinus rhythm were recorded between drivetrains, enabling comparison of sinus rhythm activation.

### Data analysis and statistics

ARIs, as an estimate of APD,^9^, ^10^, ^11^ were calculated semiautomatically by means of the Wyatt method^12^ using automated custom algorithms implemented off-line^13^ (MATLAB, MathWorks). In brief, the steepest downstroke of the unipolar electrogram was taken as an indicative of local activation time and the steepest upstroke of the unipolar T wave was taken as the repolarization time. All electrogram data were checked manually.

The absolute dispersion of repolarization for a single paced beat was measured as the time difference between the earliest measured repolarization and the latest measured repolarization time. All repolarization data were examined during RV pacing; thus, RV electrode sites tended to be the earliest to repolarize and LV epicardial sites the last.

Measurements in absolute dispersion of repolarization taken from just 2 electrode sites per patient may be susceptible to measurement error. In order to take advantage of multiple electrode sites for each patient, we confirmed trends in repolarization by measuring the standard deviation of repolarization times across all electrodes and the interquartile ranges.^14^, ^15^

Repolarization dispersion data were normalized to the mean dispersion measured during steady-state pacing during the active relaxation protocol for each patient. This allowed comparison between patients.

We have previously detailed some of the challenges in analyzing electrogram restitution data.^5^ All statistical analysis was performed with R software (version 14.0.7, The R Foundation). Multiple measurements were acquired from each electrode, each catheter itself had 8–10 electrodes, and several catheters were used in each individual patient. Multilevel regression was used to preserve the multilevel nature of data. This technique allows statistical models to be created accounting for data levels, and the probability of data groups coming from the same population of data were inferred by comparing models using analysis of variance comparisons. Dynamic comparisons of the dispersion of repolarization were performed graphically using LOESS (LOcal regrESSion) modeling to combine data sets. This nonparametric method allows trends and confidence intervals in scatterplot data acquired to be visualized. Paired single-level data were compared using the Student *t* test. Data are presented as mean ± SD unless otherwise stated in the text.

## Results

### Patient population

Sixteen patients were enrolled (12 women and 4 men; median age 34 years; range 22–64 years). Four patients did not undergo full restitution stimulation protocols, and results are presented from 12 patients (9 women and 3 men) who completed at least 1 mental stress protocol satisfactorily. Reasons for noncompletion of full protocols were lack of time (2 patients) and patient refusal to continue with nonclinical study (2 patients).

The final clinical diagnoses were atrioventricular nodal reentrant tachycardia (7 patients) and atrioventricular reentrant tachycardia (3 patients). No inducible arrhythmia occurred in 2 patients, that is, normal electrophysiological study.

### Efficacy of induction of mental stress

All 12 patients reported low stress during the relaxation protocol (2.4 ± 0.7; 1 = no stress at all and 10 = most stressed possible). All 12 patients completed the mental arithmetic protocol, and 7 patients were also able to complete the anger recall protocol. Five of these 7 patients reported greater stress during anger recall. All patients reported raised stress during mental stress protocols (mean of maximal stress 7.1 ± 0.7; *P* < .01). An increase in the sinoatrial rate from a mean of 76 ± 16 beats/min during active relaxation to 96 ± 20 beats/min during maximal stress was observed (*P* < .001). Similarly, blood pressure increased from means of 122 ± 15 mm Hg (systolic) and 71 ± 8 mm Hg (diastolic) to 135 ± 18 and 86 ± 9 mm Hg, respectively (*P* < .001, both measures).

### Steady-state ARIs are reduced during mental stress

During drivetrain pacing (S_1_) in the active relaxation state, ARIs were longer in the LV endocardium than in the epicardium (LV_Endo_ 237 ± 32 ms; LV_Epi_ 204 ± 30 ms; *P* < .001) or RV endocardium (206 ± 28 ms; *P* < .001).

**Figure 2:**
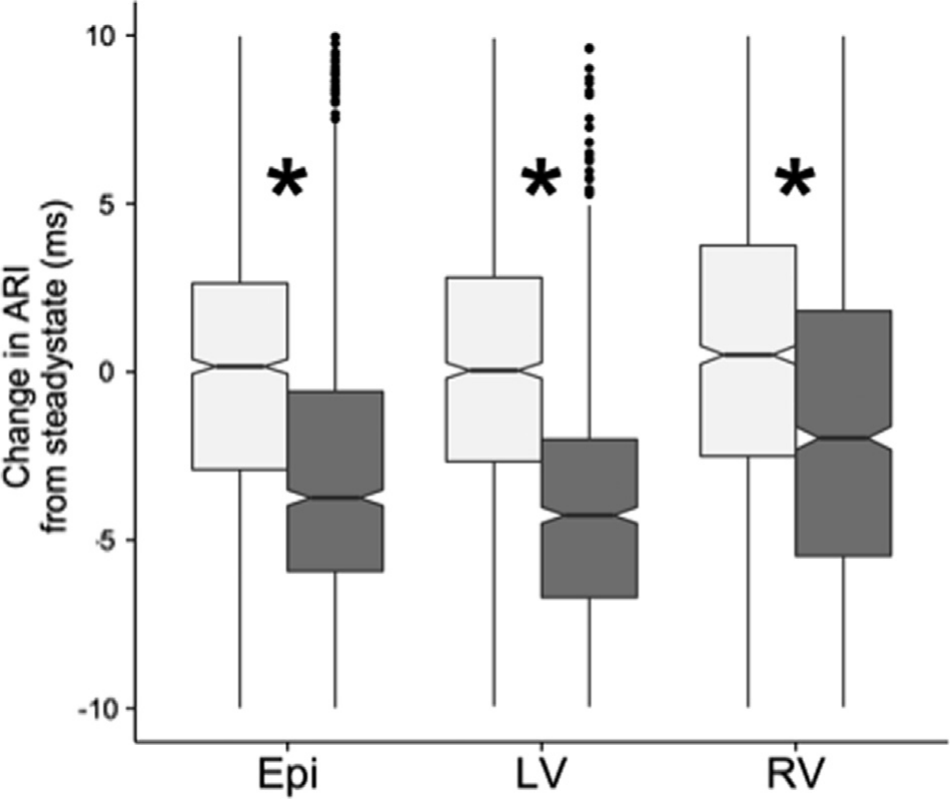
Activation-recovery interval (ARI) reductions during mental stress. A box plot of the change in ARI as compared to the mean ARI during steady-state pacing in the active relaxation protocol is shown. Light boxes indicate data acquired during relaxation, and dark boxes indicate ARI during mental stress. Asterisk represents *P* < .001 (analysis of variance multilevel regression). A significant reduction in ARI during mental stress was observed in all 3 measurement locations. Epi = epicardial; LV = left ventricular endocardium; RV = right ventricular endocardium.

ARIs were reduced during mental stress across all sites (*P* < .001), with a greater reduction in the LV endocardium than in the epicardium or RV (mean change in ARI during mental stress compared to baseline values: LV_Endo_ −8 ms; LV_Epi_ −5.0 ms; RV_Endo_ −4 ms; Figure 2).

### Restitution of ARI

**Figure 3:**
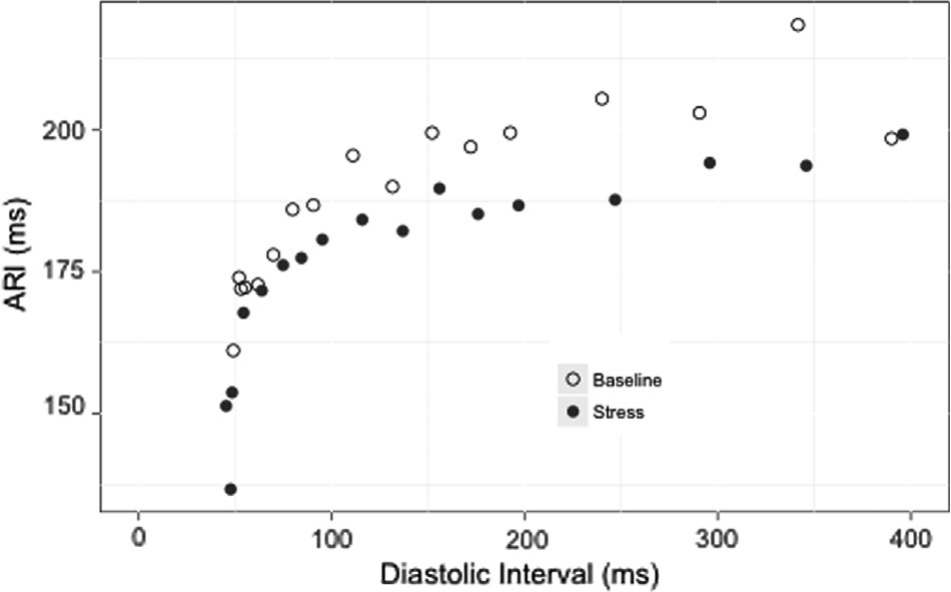
Example of dynamic activation recovery interval (ARI) restitution curve taken from the left ventricular endocardium. A single electrical restitution curve taken from the left ventricular endocardium is shown. During stress, shorter ARIs are achieved along the entire restitution curve. This is particularly evident at very short diastolic intervals, where ARIs are markedly shorter than during active relaxation.

Marked interchamber variability in restitution slopes occurred during active relaxation, with the epicardium exhibiting the shallowest restitution slope (slope 0.55; 95% confidence interval 0.46–0.64) and steeper restitution in the LV endocardium (slope 1.0; 95% confidence interval 0.81–1.3; *P* < .001) and RV (slope 0.78; 95% confidence interval 0.56–1.00; *P* < .001, all comparisons). Mental stress resulted in an overall depression of the entire electrical restitution curve. The reduction in ARI during mental stress during S_1_ pacing was maintained throughout the restitution curve (Figure 3), as were the regional variations in ARI across the ventricle. Mental stress reduced RV ERP from 228 ± 23 to 221 ± 21 (*P* < .001).

The minimum ARI values observed at short diastolic intervals (ie, the shortest left-hand ARI on the restitution curve) decreased substantially in the LV endocardium (mean ARI: LV_Endo_ 195 ± 31 at rest to 182 ± 32 during mental stress; *P* < .001), commensurate with the decrease in ERP. Less marked decreases in the minimum APDs at short coupling intervals were also observed in the LV epicardium (178 ± 27 to 175 ± 26 ms) and RV endocardium (156 ± 11 to 153 ± 12 ms). Comparisons of all ARIs at diastolic intervals within 20 ms of the shortest diastolic intervals confirmed this effect (Table 1).

**Table 1:** Comparison of all ARIs

Site	Mean ARI (ms)					
	Steady state			Short diastolic interval		
	Rest	Stress	*P*	Rest	Stress	*P*
LV endocardium	239 ± 30	231 ± 32	<.001	208 ± 18	199 ± 16	<.001
LV epicardium	206 ± 29	198 ± 26	.02	186 ± 12	173 ± 10	<.001
RV endocardium	206 ± 27	200 ± 28	<.005	165 ± 8	161 ± 13	.02

Calculated mean ARIs are shown during active relaxation and mental stress, both during steady-state pacing and at diastolic intervals within 20 ms to the minimum diastolic interval. *P* values are inferred from analysis of variance multilevel regression.

LV = left ventricular; RV = right ventricular.

Maximal restitution curve slopes (S_max_) were not significantly greater during stress in the LV endocardium or RV, but a slight increase in slope was observed in the epicardium (∂S_max_ = +0.07; 95% confidence interval 0.05–0.10; *P* < .01).

### Alterations in the dispersion of repolarization

**Figure 4:**
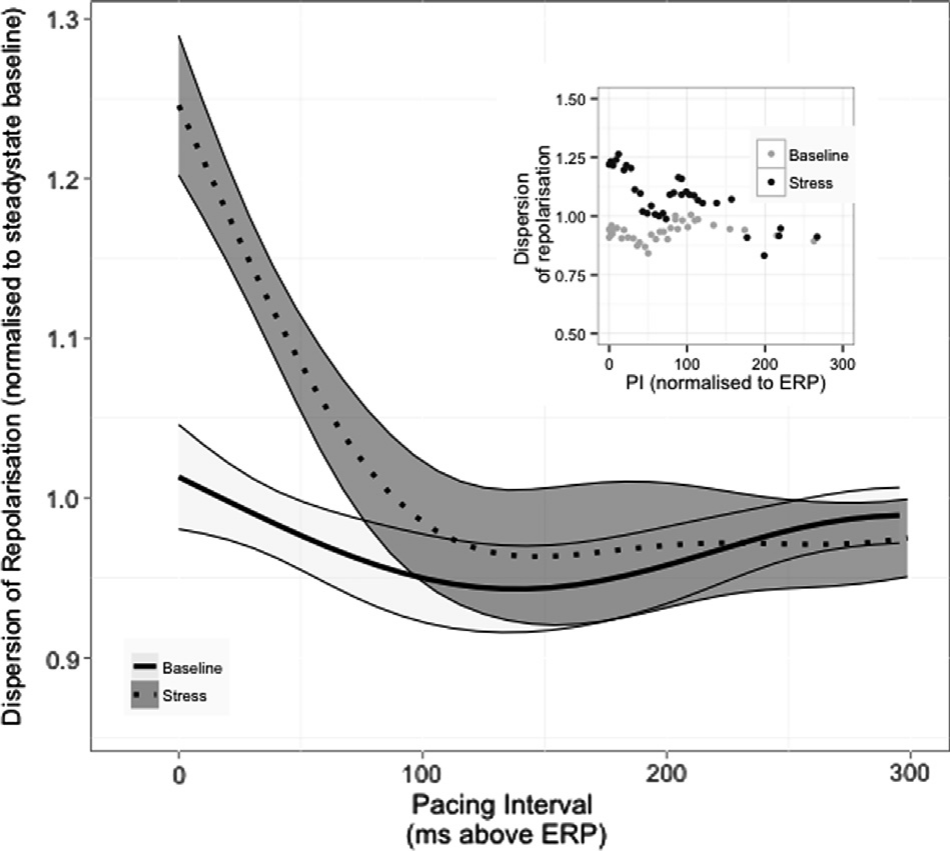
Dispersion of repolarization. The dynamics of total dispersion of repolarization obtained in 12 patients are shown, normalized to mean dispersion during steady-state pacing during active relaxation. Pacing interval (PI) is normalized to milliseconds above the effective refractory period (ERP). The shaded bands represent 95% confidence intervals, calculated using LOESS (LOcal regrESSion) regression, and separation of these bands implies statistical significance. There is a significant increase in the dispersion of repolarization during stress as pacing intervals approach effective refractory period during stress, which is not present at rest (*P* < .001; see Table 2). Representative data from 1 patient are shown in the inset.

Changes in the dynamic dispersion of repolarization in response to stress and to reductions in coupling intervals were examined. During steady-state pacing, no significant change in dispersion of repolarization was observed across all measured sites. A sharp increase in ventricular dispersion of repolarization was observed during stress at coupling intervals approaching ERP, which was not apparent during baseline relaxation (dispersion during relaxation: median 117 ms; range 63-192 ms; dispersion during stress: median 140 ms; range 83-201 ms; Figure 4). Dispersion of repolarization at longer coupling intervals showed only minor nonsignificant changes during mental stress. During mental stress, dispersion of repolarization increased at short coupling intervals to 127% ± 22% of steady-state dispersion (*P* < .001; Table 2). This was not observed during active relaxation, where no significant change in dispersion with premature beats occurred (97% ± 15%). Increases in the standard deviation of repolarization times and in the interquartile ranges of repolarization times also increased significantly (Table 2).

**Table 2:** Dynamic dispersion of repolarization

Variable	Dispersion (%)					
	Steady state			Short diastolic intervals		
	Rest	Stress	*P*	Rest	Stress	*P*
Total	100 ± 0	97 ± 15	0.97	94 ± 21	127 ± 22	<.001
SD	100 ± 0	92 ± 16	0.522	91 ± 23	122 ± 21	<.001
IQR	100 ± 0	84 ± 19	0.35	84 ± 38	113 ± 27	<.001

Three measures of dispersion of repolarization are shown: absolute dispersion of repolarization, standard deviation (SD), and interquartile range (IQR). Values are normalized to individual patient steady-state values during active relaxation and represented as percentage. *P* values are inferred from analysis of variance multilevel regression.

NS = not significant.

Apical-basal repolarization gradients were examined. The increase in RV apical-basal gradients during stress at short coupling intervals mirrored those observed across the entire ventricle with an increase in dispersion to 115% ± 56% of baseline (*P* < .001). However, absolute repolarization gradients across individual catheters were generally small (mean RV 38 ms, LV endocardium 39 ms, and LV epicardium 29 ms during active relaxation) and no consistent change in gradients were observed within the LV or epicardium during mental stress.

### Arrhythmias associated with mental stress

**Figure 5:**
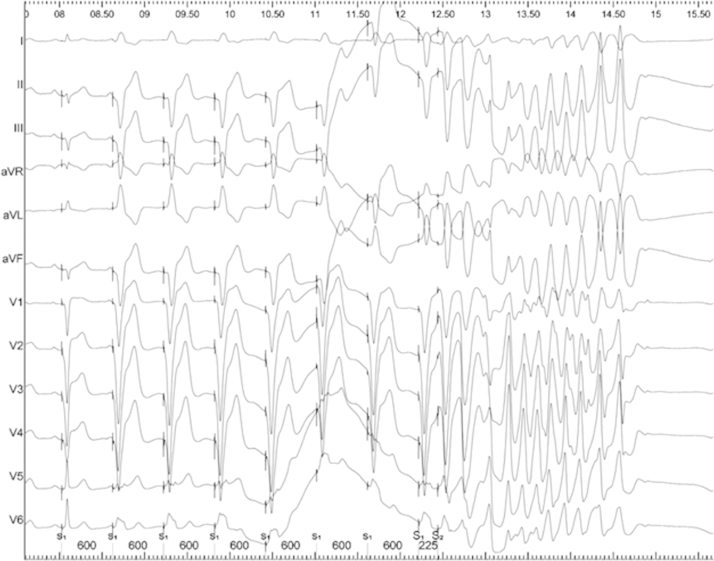
Arrhythmia induced during mental stress protocol. During the anger recall portion of the mental stress protocol in 1 patient, delivery of 1 programmed stimulus (S_2_) to the right ventricle induced 1.5 seconds of rapid polymorphic ventricular tachycardia. No arrhythmia had been induced in this patient during programmed stimulation performed in the relaxation state.

Only a single patient developed ventricular arrhythmia during mental stress (anger recall stage) following a single short-coupled S_2_ beat (Figure 5). The triggering premature ventricular contraction (PVC) has a near-identical morphology to the paced beats, suggesting that it arose close to the pacing site. The onset of the local electrogram of this beat was later than the onset of the surface depolarization, which would argue against catheter tip pressure as a triggering factor. Although this may have been a chance finding, arrhythmia did not occur during the relaxation phase at any coupling interval. No other sustained arrhythmia was observed. No arrhythmia was induced at similar coupling intervals at rest.

## Discussion

This study demonstrates that marked changes in local myocardial activation and recovery can be induced and measured in people using mental stress protocols. The key findings are as follows: (1) the cardiac action potential shortens during mental stress to differing degrees within the LV endocardium, LV epicardium, and RV; (2) these reductions in action potential length are maintained throughout the electrical restitution curve; (3) a substantial reduction in minimum ARIs at short coupling intervals is observed during mental stress; and (4) the dispersion of repolarization markedly increases at short coupling intervals during mental stress as compared to the relaxed state.

An important and novel finding is that dispersion of repolarization was markedly increased exclusively at short coupling intervals during stress. ERP was reduced during stress, as shortest-coupled S2 beats were more premature during stress than at rest. Conduction across the heart is slowed following such closely coupled extrastimuli.^13^ The cumulative effects of conduction slowing and heterogeneity in APD restitution across the heart will act to increase the dispersion of repolarization at short coupling intervals as we observed.

This resolves an apparent discrepancy in our observations; the cardiac chamber with the longest ARI (the LV endocardium) also exhibits the greatest reduction in ARI during mental stress. Although ARIs become closer across the heart during mental stress, the absolute dispersion of repolarization is also dependent on conduction across the heart. As ERP shortens, there is increased slowing of conduction at very short coupling intervals (conduction velocity restitution). The combined effects of shortening ARI near the stimulus site and increasing conduction slowing across the heart increase the repolarization dispersion of the most premature beats possible during stress. Indeed, conduction velocity restitution protects distant sites from short diastolic intervals and maximal restitution. Therefore at the shortest coupling intervals, ARI restitution acts maximally near the site of stimulation, yet distant sites never experience very short diastolic intervals. The cumulative effect will be an increase in absolute dispersion of repolarization.

Our finding of an increased apical-basal dispersion of repolarization at short coupling intervals during mental stress within the right ventricle supports this hypothesis.

The increased dispersion of repolarization provides a plausible explanation for the arrhythmogenic consequences of mental stress. In the clinical laboratory it is a common observation that tachycardia induction may hinge on coupling intervals either side of a critical window of only a few milliseconds.

The stress-induced alterations in refractoriness we observed (median 7-ms reduction in ERP) might be sufficient to enable a spontaneous ventricular ectopic beat to initiate wavebreak, reentry, and clinical arrhythmia under stress but not under resting conditions. Such an ectopic would be an isolated event during resting conditions. In our study, a single ventricular ectopic (VE) with a coupling interval of 225 ms was seen to trigger an unstable ventricular arrhythmia, but the same paced coupling interval during resting conditions had previously not been arrhythmogenic. Of course, this isolated event may be attributable to chance.

An increased spatial dispersion of repolarization has previously been shown to be arrhythmogenic in animal and computer models.^16^, ^17^ Preexisting heterogeneities in repolarization and conduction (eg, in the periscar region around an old infarct) would likely be amplified by autonomic changes, and repolarization gradients increased.

Our observation that shorter APDs occurred at short diastolic intervals is relevant to the temporal correlation of arrhythmias with mental stress. This degree of shortening of 13 ms in the LV endocardium at short coupling intervals is 1.6-fold of that observed in the steady state. Thus, the dynamic changes induced by closely coupled beats amplify mental stress effects on both APD and repolarization gradients.

The changes in ARI observed in this study were greater than those in a previous study using movie clips to induce mental stress in patients with atrial fibrillation.^6^ This may be attributed to more effective induction of mental stress, a younger patient cohort without previously frequent arrhythmias, and naivety to antiarrhythmic medications in the present study.

The minimal APD (the leftmost point on the restitution curve) is considered one of the most important factors in determining organization of the excitation pattern of ventricular fibrillation and in the stability of reentrant.^18^, ^19^ The shortened APDs at short diastolic intervals during mental stress is linked to ventricular arrhythmias being more likely to degenerate to fibrillation under stressful conditions. Thus, a premature extrasystole may be more likely to trigger ventricular fibrillation in a predisposed patient during mental stress. This is in keeping with previous observations^20^, ^21^ that ventricular arrhythmias were more easily inducible and persisted for longer time during mental stress in patients undergoing programmed electrical stimulation via implanted defibrillators.

We did not observe a marked increase in the maximal slope of APD restitution during mental stress. This contrasts with previous work from our group,^5^ where a significant increase in restitution slope during isoprenaline administration in humans was observed. Our finding may be accounted for by the induction of more subtle increases in intrinsic sympathetic activity through mental stress vs the use of incremental isoprenaline and adrenaline infusions. Whereas administration of sympathomimetic drugs can be expected to have a global effect on cardiac electrophysiology, the highly regional and complex innervation of the cardiac autonomics can give rise to functional asymmetry during cardiac autonomic stimulation.^22^ Intrinsic autonomic stimulation is expected to involve both parasympathetic and sympathetic components, whose multifaceted effects may be simultaneously complimentary and divergent. While pure adrenergic stimulation may shorten APD and steepen restitution curves, the alterations in sympathetic and parasympathetic balance may allow restitution curves not to steepen at short coupling intervals.

The exact reasons underlying differences in restitution between cardiac chambers are not clear, and several mechanisms could be involved. These include regional variability in ion channel expression and function^23^, ^24^ and local interaction with mechanoelectric feedback in response to sympathetically mediated changes in myocardial contraction,^25^ as well as regional differences in autonomic innervation, which could modulate both ARI and maximal restitution slope.^26^ Whatever the underlying mechanisms, regional differences in repolarization and hence refractoriness are likely to be proarrhythmic by facilitating functional block and reentry.

### Strengths and limitations

This study is the first to directly assess dynamic human cardiac electrophysiology during mental stress induction. A particular strength is that it was performed in individuals with essentially normal hearts and without medication. It thus provides an important benchmark of the normal electrophysiological response to mental stress in humans. However, further study is necessary to confirm that these effects are critical in patients with known predisposition to arrhythmia.

Several limitations exist. First, the invasive procedure used to measure cardiac electrophysiology is liable to cause anxiety in itself. Anxiolytic agents would of course be liable to affect the results of the study and make stress induction difficult or impossible. Although the active relaxation protocol was chosen specifically to address this, it is likely a background anxiety remained. Ideally a measure of sympathetic stress response would have been included, for example, serum catetcholamines, and this was not performed. However, this would not have influenced the interpretation of our findings.

The mental arithmetic protocol was followed by anger recall; the more structured and impersonal nature of the arithmetic protocol was found to be more reliably administered during the invasive electrophysiological study during “test runs” and hence the order chosen. However, had it been possible to administer anger recall to all patients, higher maximal stress would have occurred.

The measurement of within-chamber dispersion of repolarization was limited by the relatively small area of myocardium covered in either the LV endocardium or the LV epicardium. Our study was performed without global high-resolution 3-dimensional mapping technology, which would certainly aid examination of endocardial apical-basal repolarization gradients. This may be addressed more fully in a future study.

We did not expect arrhythmia to occur as a result of our protocol during the study. Although the observation of an episode of (nonsustained) polymorphic ventricular tachycardia in 1 patient during the stress protocol lends credence to the assertion that mental stress can be proarrhythmic, this single instance can only be viewed as being circumstantial evidence, since neither its reproducibility nor its generalizability could be assessed.

## Conclusion

Conscious mental stress induces measurable and significant changes in dynamic electrophysiology across the human heart. These changes may underlie the mechanism of the promotion of arrhythmia during mental stress.

**Clinical Perspectives**

This article describes the significant reductions in action potential durations that can be induced in the human heart by mental stress. This was measured directly during invasive electrophysiological studies in patients with essentially normal hearts and free of medication. We demonstrate for the first time that interactions between this and closely coupled premature extrastimuli can lead to markedly increased dispersion of repolarization during stress. This provides a fertile proarrhythmic substrate that may explain the promotion of arrhythmia by mental stress. Knowledge of these dynamics helps us understand the temporal relation of arrhythmia to external conscious stimuli and provides a mechanism by which neurostimulation and modulation might be helpful as a therapeutic tool. Future studies will better define whether such mechanisms can be directly harnessed in patients with underlying cardiac disease to treat and prevent arrhythmia.
